# The national utilization of nonoperative management for small renal masses over 10 years

**DOI:** 10.1093/jncics/pkad084

**Published:** 2023-10-06

**Authors:** Dejan K Filipas, Edoardo Beatrici, Jose I Nolazco, Zhiyu Qian, Phillip Marks, Muhieddine Labban, Benjamin V Stone, Phillip M Pierorazio, Stuart R Lipsitz, Quoc-Dien Trinh, Steven L Chang, Alexander P Cole

**Affiliations:** Department of Urology and Center for Surgery and Public Health, Brigham and Women’s Hospital, Boston, MA, USA; Department of Urology, University Medical Center Hamburg-Eppendorf, Hamburg, Germany; Department of Urology and Center for Surgery and Public Health, Brigham and Women’s Hospital, Boston, MA, USA; Department of Urology and Center for Surgery and Public Health, Brigham and Women’s Hospital, Boston, MA, USA; Department of Urology and Center for Surgery and Public Health, Brigham and Women’s Hospital, Boston, MA, USA; Department of Urology, University Medical Center Hamburg-Eppendorf, Hamburg, Germany; Department of Urology and Center for Surgery and Public Health, Brigham and Women’s Hospital, Boston, MA, USA; Department of Urology and Center for Surgery and Public Health, Brigham and Women’s Hospital, Boston, MA, USA; Division of Urology, University of Pennsylvania, Philadelphia, PA, USA; Department of Urology and Center for Surgery and Public Health, Brigham and Women’s Hospital, Boston, MA, USA; Department of Urology and Center for Surgery and Public Health, Brigham and Women’s Hospital, Boston, MA, USA; Department of Urology and Center for Surgery and Public Health, Brigham and Women’s Hospital, Boston, MA, USA; Department of Urology and Center for Surgery and Public Health, Brigham and Women’s Hospital, Boston, MA, USA

## Abstract

**Background:**

Management of small renal masses often involves a nonoperative approach, but there is a paucity of information about the use and associated predictors of such approaches. This study aimed to determine the trends in and predictors of use of nonoperative management of small renal masses.

**Methods:**

Using data from the National Cancer Database for localized small renal masses (N0/M0, cT1a) diagnosed between 2010 and 2020, we conducted a cross-sectional study. Nonoperative management was defined as expectant management (active surveillance or watchful waiting) or focal ablation. Adjusted odds ratios (AORs) were calculated using multivariable logistic regression models.

**Results:**

Of the 156 734 patients included, 10.5% underwent expectant management, and 13.9% underwent focal ablation. Later year of diagnosis was associated with a higher likelihood of nonoperative management. In 2020, the odds of receiving expectant management and focal ablation were 90% (AOR = 1.90, 95% confidence interval [CI] = 1.71 to 2.11) and 44% (AOR = 1.44, 95% CI = 1.31 to 1.57) higher, respectively, than in 2010. Black patients had increased odds of expectant management (AOR = 1.47, 95% CI = 1.39 to 1.55) but decreased odds of focal ablation (AOR = 0.93, 95% CI = 0.88 to 0.99).

**Conclusion:**

Over the decade, the use nonoperative management of small renal masses increased, with expectant management more frequently used than focal ablation among Black patients. Possible explanations include race-based differences in physicians' risk assessments and resource allocation. Adjusting for Black race in calculations for glomerular filtration rate could influence the differential uptake of these techniques through deflated glomerular filtration rate calculations. These findings highlight the need for research and policies to ensure equitable use of less invasive treatments in small renal masses.

Renal cancer is among the most common cancers in the United States, with 80 000 new cases estimated to be diagnosed in 2023 (1). Despite a shift toward earlier detection of small renal masses over recent decades, renal cancer mortality rates have persisted without improvement, leading to concerns about the possibility of overdiagnosis and overtreatment (2). Due to the low risk of progression, clinical guidelines incorporated a nonoperative management option for small renal masses (≤4 cm) into the National Comprehensive Cancer Network and American Urological Association guidelines in 2009, followed by the European Association of Urology guidelines in 2010 (3-7). Nonoperative management has since become a valuable alternative in select patients (8). Although surgery for suspicious renal masses is safe for even complex renal masses, a small number of individuals may have severe complications, and nonoperative management can decrease the burden of unnecessary surgery on patients with small renal masses (9-11). Nonoperative interventions are available in a spectrum of approaches, from entirely nephron-sparing strategies such as active surveillance to those that may inadvertently affect healthy renal tissue, such as focal ablation. Nephron-sparing strategies are imperative in circumstances necessitating renal function preservation, particularly in the context of a diminished estimated glomerular filtration rate (GFR).

Although patient-level variables (such as age, race, GFR, and comorbidities) may affect the use of surgery vs nonoperative management, institutional practice patterns, health systems factors, and time trends may also play a role (12). In this setting, we designed a study to assess how the year of diagnosis and nonclinical patient factors may affect the likelihood of nonoperative management. We focused on both expectant management (surveillance) and ablative therapies.

## Methods

We followed the Strengthening the Reporting of Observational Studies in Epidemiology reporting guidelines for cross-sectional studies (13). This study was approved by the institutional review board of Brigham and Women’s Hospital (protocol No. 2016P000517).

### Data source

We queried data from the National Cancer Database (NCDB), a comprehensive cancer registry established by the Commission on Cancer of the American Cancer Society. This database comprises 1500 Commission on Cancer–accredited hospitals and covers a substantial number of cancer cases in the United States, including 86.7% of all renal cancer cases (14). Trained data abstractors gathered sociodemographic and medical information from participating institutions using a standardized approach. The database systematically collects clinical, pathologic, and treatment data.

### Study population

Men and women with newly diagnosed renal masses from 2010 to 2020 within the NCDB were included in this study. Our analytical cohort consisted of patients with localized (N0/M0) small renal masses (cT1a).

### Outcome measure and covariates

Our primary outcome was the receipt of nonoperative management, which consisted either of expectant management (active surveillance or watchful waiting) or focal ablation (predominantly cryosurgery or thermal ablation). *Nonoperative management* refers to any treatment option that does not involve surgical intervention. Focal ablation can be considered a type of nonoperative management for renal masses. It is a minimally invasive alternative to surgery that does not involve removing the tumor through traditional surgical methods. We used the variables *rx_summ_treatment_status* and *rx_surg_prim_site.* Patients coded with active surveillance and no surgery of the primary site were considered for expectant management. The covariates included were age at diagnosis, gender, comorbidities, insurance status, income, education level, type of and distance to a facility, and race or ethnicity, as provided by the NCDB. We excluded races other than White and Black from our analytical cohort due to the low patient count. Comorbidities were coded based on *International Classification of Diseases, Ninth Revision/International Statistical Classification of Diseases, Tenth Revision* codes and weighted according to the Charlson-Deyo score, a modification of the Charlson Comorbidity Index (15).

### Statistical analysis

We used descriptive statistics to compare patients receiving nonoperative management. Baseline characteristics were compared with Pearson χ^2^ test for categorical and standardized differences for continuous variables. We modeled 3 multivariable logistic regression models accounting for the above-mentioned covariates to estimate the adjusted odds ratio (AOR) of receiving nonoperative management, expectant management, or focal ablation (16). To control for multiple tests, we applied the false discovery rate correction, and the *P* values presented are Benjamini-Hochberg adjusted (17). With the unique circumstances that the COVID-19 pandemic presented to the health-care system starting in early 2020, we added an adjusted model for the prepandemic (2018-2019) and pandemic (2020) periods. All statistical analyses were performed using Stata, release 17 (StataCorp LP, College Station, TX). The *P* values reported are 2-sided, and *P *under .05 was considered statistically significant.

## Results

A total of 156 734 patients met the inclusion criteria. Of those included, 60.7% were male, 86% were White, 10.5% received expectant management, and 13.9% received focal ablation (Supplementary Figure 1, available online). The median age was 63 years (interquartile range = 54-71 years). There was a shift in proportion toward the older age groups from 2010 to 2020 (*P* < .001), with the median (interquartile range) age in 2010 being 63 (53-71) years and in 2020 being 64 (55-72) years. A shift in proportion toward a sicker patient population was observed, with the proportion of 2 or more comorbidities increasing from 9.55% in 2010 to 17.28% in 2020 (*P* < .001). A detailed description of baseline characteristic by year is provided in Table 1.

**Table 1.** Baseline characteristics of patients with small renal masses inside the National Cancer Database between 2010 and 2020

	2010, No. (%)	2011, No. (%)	2012, No. (%)	2013, No. (%)	2014, No. (%)	2015, No. (%)	2016, No. (%)	2017, No. (%)	2018, No. (%)	2019, No. (%)	2020, No. (%)	Total, No. (%)	***P*** ^a^
	**N = 10** **255**	**N = 10** **720**	**N = 11** **620**	**N = 12** **381**	**N = 13** **500**	**N = 14** **858**	**N = 16** **333**	**N = 17** **226**	**N = 17** **214**	**N = 17** **803**	**N = 14** **824**	**N = 156** **734**	
Age at diagnosis, y													.001
≤49	1841 (18.0)	1927 (18.0)	1961 (16.9)	2037 (16.5)	2277 (16.9)	2487 (16.7)	2645 (16.2)	2659 (15.4)	2684 (15.6)	2692 (15.1)	2310 (15.6)	25 520 (16.3)	
50-59	2368 (23.1)	2519 (23.5)	2788 (24.0)	2827 (22.8)	3177 (23.5)	3419 (23.0)	3842 (23.5)	3761 (21.8)	3733 (21.7)	3641 (20.5)	3073 (20.7)	35 148 (22.4)	
60-69	2976 (29.0)	3162 (29.5)	3461 (29.8)	3726 (30.1)	4154 (30.8)	4652 (31.3)	5161 (31.6)	5421 (31.5)	5433 (31.6)	5651 (31.7)	4625 (31.2)	48 422 (30.9)	
70-79	2216 (21.6)	2183 (20.4)	2504 (21.5)	2737 (22.1)	2866 (21.2)	3217 (21.7)	3417 (20.9)	3995 (23.2)	4031 (23.4)	4373 (24.6)	3688 (24.9)	35 227 (22.5)	
≥80	854 (8.3)	929 (8.7)	906 (7.8)	1054 (8.5)	1026 (7.6)	1083 (7.3)	1268 (7.8)	1390 (8.1)	1333 (7.7)	1446 (8.1)	1128 (7.6)	12 417 (7.9)	
Sex													.001
Male	6181 (60.3)	6364 (59.4)	6936 (59.7)	7445 (60.1)	8164 (60.5)	8960 (60.3)	9966 (61.0)	10 488 (60.9)	10 553 (61.3)	10 939 (61.4)	9169 (61.9)	95 165 (60.7)	
Female	4074 (39.7)	4356 (40.6)	4684 (40.3)	4936 (39.9)	5336 (39.5)	5898 (39.7)	6367 (39.0)	6738 (39.1)	6661 (38.7)	6864 (38.6)	5655 (38.1)	61 569 (39.3)	
Race													.005
Black	1259 (12.8)	1396 (13.5)	1541 (13.8)	1669 (14.1)	1813 (14.0)	1988 (14.0)	2199 (14.2)	2298 (14.0)	2252 (13.8)	2506 (14.9)	1915 (13.7)	20 836 (14.0)	
White	8556 (87.2)	8908 (86.5)	9621 (86.2)	10 194 (85.9)	11 102 (86.0)	12 194 (86.0)	13 328 (85.8)	14 073 (86.0)	14 009 (86.2)	14 357 (85.1)	12 058 (86.3)	128 400 (86.0)	
Median income quartile													.001
$74 063 or more	3155 (30.8)	3372 (31.5)	3450 (29.7)	3803 (30.7)	3923 (29.1)	4523 (30.4)	4868 (29.8)	5104 (29.6)	5125 (29.8)	5236 (29.4)	4157 (28.0)	46 716 (29.8)	
$57 857 to $74 062	2196 (21.4)	2242 (20.9)	2450 (21.1)	2526 (20.4)	2730 (20.2)	3041 (20.5)	3268 (20.0)	3411 (19.8)	3406 (19.8)	3502 (19.7)	3042 (20.5)	31 814 (20.3)	
$46 277 to $57 856	2033 (19.8)	2157 (20.1)	2326 (20.0)	2442 (19.7)	2569 (19.0)	2816 (19.0)	3115 (19.1)	3209 (18.6)	3197 (18.6)	3364 (18.9)	2825 (19.1)	30 053 (19.2)	
Less than $46 277	1604 (15.6)	1633 (15.2)	1897 (16.3)	1922 (15.5)	2165 (16.0)	2244 (15.1)	2458 (15.0)	2571 (14.9)	2558 (14.9)	2628 (14.8)	2193 (14.8)	23 873 (15.2)	
Unknown	1267 (12.4)	1316 (12.3)	1497 (12.9)	1688 (13.6)	2113 (15.7)	2234 (15.0)	2624 (16.1)	2931 (17.0)	2928 (17.0)	3073 (17.3)	2607 (17.6)	24 278 (15.5)	
Primary payor													.001
Private	4555 (44.4)	4747 (44.3)	4931 (42.4)	5204 (42.0)	5591 (41.4)	6179 (41.6)	6693 (41.0)	6838 (39.7)	6727 (39.1)	6660 (37.4)	5614 (37.9)	63 739 (40.7)	
Medicare	4598 (44.8)	4831 (45.1)	5364 (46.2)	5770 (46.6)	6319 (46.8)	6867 (46.2)	7675 (47.0)	8332 (48.4)	8354 (48.5)	8925 (50.1)	7355 (49.6)	74 390 (47.5)	
Medicaid	552 (5.4)	581 (5.4)	648 (5.6)	686 (5.5)	915 (6.8)	1023 (6.9)	1198 (7.3)	1205 (7.0)	1306 (7.6)	1288 (7.2)	1087 (7.3)	10 489 (6.7)	
Other/uninsured/unknown	550 (5.4)	561 (5.2)	677 (5.8)	721 (5.8)	675 (5.0)	789 (5.3)	767 (4.7)	851 (4.9)	827 (4.8)	930 (5.2)	768 (5.2)	8116 (5.2)	
Nonoperative management													.001
No	8139 (81.9)	8492 (81.7)	9086 (80.6)	9398 (78.7)	10 265 (78.4)	11 114 (77.3)	12 039 (76.1)	12 591 (75.3)	12 492 (74.7)	12 609 (72.9)	10 479 (72.8)	116 704 (76.8)	
Yes	1801 (18.1)	1898 (18.3)	2193 (19.4)	2539 (21.3)	2827 (21.6)	3273 (22.7)	3774 (23.9)	4140 (24.7)	4232 (25.3)	4697 (27.1)	3912 (27.2)	35 286 (23.2)	
Expectant management													.001
No	9515 (92.8)	9892 (92.3)	10 626 (91.4)	11 124 (89.8)	12 116 (89.7)	13 345 (89.8)	14 542 (89.0)	15 321 (88.9)	15 207 (88.3)	15 539 (87.3)	12 997 (87.7)	140 224 (89.5)	
Yes	740 (7.2)	828 (7.7)	994 (8.6)	1257 (10.2)	1384 (10.3)	1513 (10.2)	1791 (11.0)	1905 (11.1)	2007 (11.7)	2264 (12.7)	1827 (12.3)	16 510 (10.5)	
Ablation													.001
No	8139 (88.5)	8492 (88.8)	9086 (88.3)	9398 (88.0)	10 265 (87.7)	11 114 (86.3)	12 039 (85.9)	12 591 (84.9)	12 492 (84.9)	12 609 (83.8)	10 479 (83.4)	116 704 (86.1)	
Yes	1061 (11.5)	1070 (11.2)	1199 (11.7)	1282 (12.0)	1443 (12.3)	1760 (13.7)	1983 (14.1)	2235 (15.1)	2225 (15.1)	2433 (16.2)	2085 (16.6)	18 776 (13.9)	
Charlson-Deyo score													.001
0	6889 (67.2)	7262 (67.7)	7795 (67.1)	8316 (67.2)	9026 (66.9)	10 061 (67.7)	10 937 (67.0)	11 523 (66.9)	11 321 (65.8)	11 758 (66.0)	9675 (65.3)	104 563 (66.7)	
1	2386 (23.3)	2433 (22.7)	2698 (23.2)	2859 (23.1)	3217 (23.8)	3190 (21.5)	2933 (18.0)	3029 (17.6)	2956 (17.2)	3062 (17.2)	2587 (17.5)	31 350 (20.0)	
2	980 (9.6)	1025 (9.6)	1127 (9.7)	1206 (9.7)	1257 (9.3)	1607 (10.8)	2463 (15.1)	2674 (15.5)	2937 (17.1)	2983 (16.8)	2562 (17.3)	20 821 (13.3)	

a Benjamin-Hochberg adjusted *P* values for multiple comparisons using false discovery rate correction for multiple comparisons.

### Multivariable logistic regression

In our multivariable logistic regression model for nonoperative management, Black race and ethnicity was associated with increased odds of nonoperative management (AOR = 1.17, 95% confidence interval [CI] = 1.12 to 1.22). The adjusted odds of any type of nonoperative management (either expectant management or surveillance) in 2020 was 67% higher (AOR = 1.67, 95% CI = 1.55 to 1.79) than in 2010.

In our model for expectant management, we found that Black race was associated with increased odds of receiving expectant management (AOR = 1.47, 95% CI = 1.39 to 1.55). The adjusted odds of expectant management increased yearly, being statistically significant from 2012. The odds in 2020 were 90% higher (AOR = 1.90, 95% CI = 1.71 to 2.11) than in 2010.

**Figure 1.**
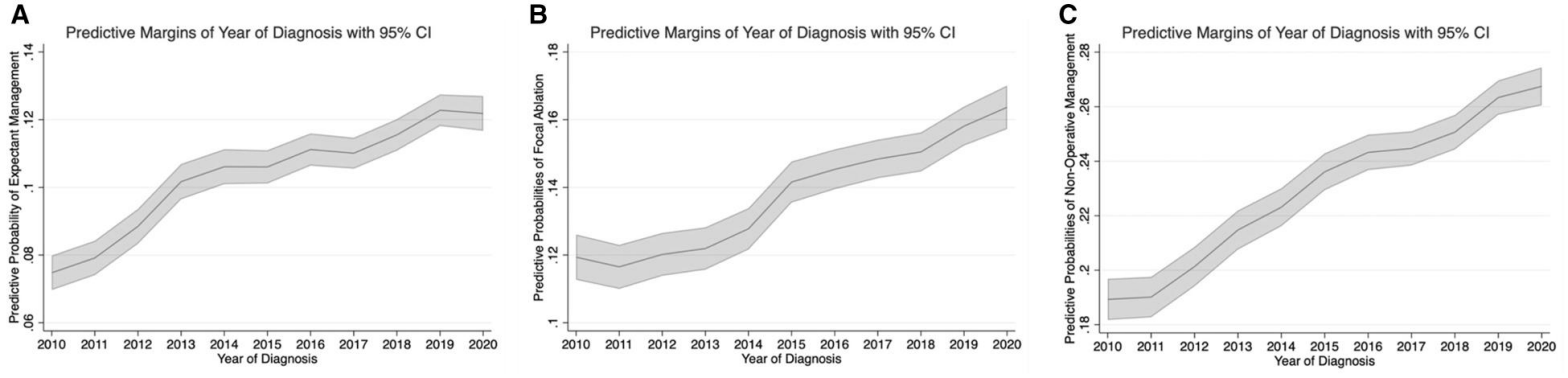
Adjusted predictive probabilities of **A**) expectant management, **B**) focal ablation, and **C**) nonoperative management from 2010 to 2020 inside the National Cancer Database. CI = confidence interval.

In our model for focal ablation, we found that the year of diagnosis was associated with increased odds of receiving focal ablation. Black race was associated with lower odds of focal ablation (AOR = 0.93, 95% CI = 0.88 to 0.99). The odds in 2020 were 44% higher (AOR = 1.44, 95% CI = 1.31 to 1.57) than in 2010. Independent predictors of both expectant management and focal ablation are shown in Figure 1 and Table 2.

**Table 2.** Multivariable logistic regression estimating the odds of expectant management, focal ablation, and nonoperative management

Covariate	**Odds Ratio** ^a^ **(95% CI)**	***P*** ^b^	**Odds Ratio** ^c^ **(95% CI)**	***P*** ^b^	**Odds Ratio** ^d^ **(95%CI)**	***P*** ^b^
Age category, y						
≤49 (Referent)	1.00	—	1.00	—	1.00	—
50-59	1.54 (1.39 to 1.70)	<.001	1.53 (1.41 to 1.67)	<.001	1.56 (1.46 to 1.67)	<.001
60-69	2.11 (1.92 to 2.33)	<.001	2.23 (2.06 to 2.42)	<.001	2.27 (2.13 to 2.42)	<.001
70-79	3.69 (3.34 to 4.08)	<.001	3.55 (3.25 to 3.87)	<.001	3.93 (3.67 to 4.21)	<.001
≥80	13.04 (11.75 to 14.46)	<.001	6.94 (6.29 to 7.65)	<.001	12.42 (11.50 to 13.40)	<.001
Charlson-Deyo score						
0 (Referent)	1.00	—	1.00	—	1.00	—
1	0.85 (0.81 to 0.89)	<.001	0.92 (0.88 to 0.97)	.001	0.88 (0.85 to 0.92)	<.001
2	1.02 (0.96 to 1.07)	.570	1.03 (0.98 to 1.08)	.377	1.04 (0.99 to 1.08)	.099
Sex						
Male (Referent)	1.00	—	1.00	—	1.00	—
Female	0.95 (0.91 to 0.99)	.013	0.87 (0.84 to 0.90)	<.001	0.89 (0.86 to 0.92)	<.001
Payor						
Private (Referent)	1.00	—	1.00	—	1.00	—
Medicare	1.42 (1.35 to 1.50)	<.001	1.36 (1.29 to 1.43)	<.001	1.41 (1.36 to 1.47)	<.001
Medicaid	1.81 (1.66 to 1.98)	<.001	1.34 (1.23 to 1.47)	<.001	1.58 (1.48 to 1.69)	<.001
Other, including uninsured and unknown	1.76 (1.61 to 1.94)	<.001	1.38 (1.26 to 1.51)	<.001	1.60 (1.49 to 1.71)	<.001
Income						
$74 063 or more (Referent)	1.00	—	1.00	—	1.00	—
$57 857 to $74 062	1.08 (1.02 to 1.14)	.006	1.19 (1.13 to 1.25)	<.001	1.16 (1.11 to 1.20)	<.001
$46 277 to $57 856	1.10 (1.03 to 1.17)	.003	1.16 (1.10 to 1.23)	<.001	1.15 (1.10 to 1.20)	<.001
Less than $46 277	1.14 (1.06 to 1.22)	<.001	1.19 (1.11 to 1.27)	<.001	1.19 (1.13 to 1.26)	<.001
Unknown	0.95 (0.64 to 1.43)	.814	1.05 (0.75 to 1.48)	.876	1.04 (0.78 to 1.38)	.812
Year of diagnosis						
2010 (Referent)	1.00	—	1.00	—	1.00	—
2011	1.07 (0.95 to 1.20)	.313	0.96 (0.87 to 1.07)	.558	1.00 (0.92 to 1.08)	.967
2012	1.20 (1.07 to 1.34)	.002	1.00 (0.91 to 1.10)	.994	1.07 (0.99 to 1.16)	.099
2013	1.45 (1.30 to 1.61)	<.001	1.00 (0.90 to 1.10)	.981	1.17 (1.09 to 1.27)	<.001
2014	1.53 (1.38 to 1.71)	<.001	1.04 (0.95 to 1.15)	.501	1.23 (1.14 to 1.33)	<.001
2015	1.52 (1.37 to 1.69)	<.001	1.15 (1.05 to 1.26)	.004	1.31 (1.22 to 1.42)	<.001
2016	1.62 (1.46 to 1.79)	<.001	1.22 (1.12 to 1.34)	<.001	1.40 (1.30 to 1.51)	<.001
2017	1.61 (1.45 to 1.78)	<.001	1.25 (1.14 to 1.37)	<.001	1.42 (1.32 to 1.52)	<.001
2018	1.71 (1.55 to 1.90)	<.001	1.26 (1.16 to 1.38)	<.001	1.46 (1.36 to 1.57)	<.001
2019	1.90 (1.72 to 2.10)	<.001	1.35 (1.24 to 1.48)	<.001	1.60 (1.49 to 1.72)	<.001
2020	1.90 (1.71 to 2.11)	<.001	1.44 (1.31 to 1.57)	<.001	1.67 (1.55 to 1.79)	<.001
Race						
White (Referent)	1.00	—	1.00	—	1.00	—
Black	1.47 (1.39 to 1.55)	<.001	0.93 (0.88 to 0.99)	.021	1.17 (1.12 to 1.22)	<.001
Facility type						
Community cancer program (Referent)	1.00	—	1.00	—	1.00	—
Comprehensive community cancer program	0.67 (0.62 to 0.73)	<.001	1.31 (1.19 to 1.44)	<.001	0.95 (0.89 to 1.02)	.183
Academic/research program	0.86 (0.79 to 0.93)	<.001	1.04 (0.95 to 1.14)	.501	0.92 (0.86 to 0.98)	.019
Integrated network cancer program	0.57 (0.52 to 0.63)	<.001	1.18 (1.07 to 1.30)	.002	0.83 (0.77 to 0.89)	<.001
Distance to hospital, mi						
0-12.4 (Referent)	1.00	—	1.00	—	1.00	—
12.5-49.9	0.73 (0.70 to 0.76)	<.001	0.96 (0.92 to 0.99)	.035	0.84 (0.81 to 0.87)	<.001
≥50	0.68 (0.63 to 0.72)	<.001	0.99 (0.94 to 1.05)	.894	0.83 (0.79 to 0.87)	<.001
Education level						
Highest >15.3% (Referent)	—	—	—	—	—	—
9.1%-15.2%	0.92 (0.87 to 0.98)	.005	1.04 (0.98 to 1.09)	.275	0.98 (0.94 to 1.02)	.303
5%-9%	0.89 (0.83 to 0.95)	<.001	1.01 (0.95 to 1.07)	.926	0.94 (0.90 to 0.98)	.013
Lowest <5%	0.85 (0.79 to 0.92)	<.001	1.09 (1.02 to 1.17)	.021	0.97 (0.92 to 1.03)	.342

a Expectant management. CI = confidence interval.

b Benjamin-Hochberg–adjusted *P* values for multiple comparisons using false discovery rate correction for multiple comparisons.

c Focal ablation.

d Nonoperative management.

### COVID-19

There was a higher proportion of focal ablation during the COVID-19 pandemic than in the prepandemic period (AOR = 1.09, 95% CI = 1.02 to 1.17). There was no statistically significant increase in the use of expectant management during the pandemic (AOR = 1.05, 95% CI = 0.98 to 1.12).

## Discussion

There was an increase in the utilization of nonoperative management across our 10-year study period. The odds of expectant management in 2020 were 90% higher than in 2010. The odds for focal ablation in 2020 were 44% higher than in 2010. Interestingly, there were race-based differences in the odds of nonoperative management, with apparently opposite effects: Although the adjusted odds of expectant management were more common among Black men and women, the odds of receiving focal ablation were lower in this group.

The rising utilization of expectant management in small renal masses can reasonably be attributed to its inclusion in international guidelines (5,18). In addition to mature data on progression, overall survival, improved quality, and widespread availability of cross-sectional imaging could have helped physicians’ confidence in delaying surgical treatment (19). For example, it has been shown that patients older than 75 years of age did not compromise on overall survival and cardiovascular adverse events when expectant management was compared with surgical options. It is noteworthy to address the between-hospital variability in the use of expectant management in the United States that was not fully explained by clinical and pathologic factors (12). New cross-sectional imaging tools, however, have reduced the need for surgery on potentially benign renal masses but have not eliminated the need for surgery because these tools have limited accuracy (20,21). We may hypothesize that the increase in accuracy and availability of high-quality cross-sectional imaging tools in part contributed to the uptake of surveillance strategies. Expectant management may be an effective option for small renal masses, particularly those smaller than 2 cm in size, with low progression rates, warranting further investigation across all age groups (7,22).

First, there is the aspect of comorbidities that can potentially divert patients from a higher-risk intervention, such as partial nephrectomy, to a less risky alternative, such as radical nephrectomy or perhaps focal ablation. Concurrently, a GFR-related factor may sway patients and health-care professionals towards increasingly nephron-sparing treatments. At this juncture, it is important to remember that nonoperative interventions offer a spectrum of approaches. They range from entirely nephron-sparing strategies, such as expectant management, to those that may inadvertently affect healthy renal tissue, such as focal ablation. This spectrum provides options yet also adds complexity to decision making. Although both nonoperative approaches shield patients from surgical complications, ablation potentially affects GFR through collateral destruction of nephrons, whereas surveillance or expectant management would not.

Focal ablation for renal cancer increased to statistically significant levels in 2015, following inclusion in international guidelines (3-5,23). This is a valuable alternative to surgery, mainly when competing comorbidities prevent surgery or patient preferences for intervention do not allow a surveillance approach. Further research is needed, however, comparing focal ablation with surgical options based on concerns about local recurrence (24,25).

The finding of racial differences in surveillance for small renal masses is novel but aligns with the existing literature on racial disparities in active surveillance in prostate cancer (26,27). Although both expectant management and ablation are comparatively modern and “less intensive” treatments for individuals with small renal masses, ablative approaches are presumably more resource intensive and have higher associated cost than surveillance. Ablation was less common among Black men and women, but expectant management was more common. Lower availability of the technology required for ablation is 1 possible explanation—research by our team and others has shown systematic differences in the hospitals that treat patients from minority groups (28,29). Other possible explanations include the possibility that Black men and women with renal cancer could be judged (rightly or wrongly) to have worse comorbidities and, therefore, greater competing risks than White patients. This finding could be the result of race-based differences in medical comorbidities such as diabetes, obesity, or hypertension or because of physician bias (30,31).

Although our findings have been adjusted for comorbidities and demographic covariates, we cannot account for patient preferences, which leads us to hypothesize that patients of Black race or ethnicity may prefer alternative options, such as expectant management or surgery (32). If this preference does not hold for small renal masses, structural barriers to ablative therapies not captured in the NCDB may exist (33). In their systematic review, Beyer et al. described various factors influencing treatment decision making in localized renal cancer. Although health-care professionals’ and patients’ decision-making criteria are more obvious, contextual factors, such as access to high-quality health care, also play a significant clinical role. Our findings align with this, as patients with private insurance are more likely to undergo operative management. Although the proportion of privately insured patients has declined over the decade, private insurance remains a predictor of operative management. These factors may contribute to Black patients being less likely to receive ablation for small renal masses but more likely to receive active surveillance (33).

From a practical standpoint, nonoperative management has gradually gained acceptance as a treatment option for small renal masses. Further research is needed to investigate the racial disparities in the utilization of ablation for small renal masses and to compare the mid- to long-term outcomes with the standard of care. Although both surveillance and ablative therapies are less intensive treatment options for small renal masses, the differential adoption of these 2 techniques by patient race is suggestive of ways in which costs and resource requirements rather than clinicodemographic variables may spur the dissemination of these tools.

This study has some noteworthy limitations. First, the NCDB is limited in analyzing trends because it only reports proportions and cannot provide reliable data on follow-up imaging, tumor progression, or missed appointments for active surveillance. The complexity of renal masses is not measured; only the diameter is reported, failing to assess another layer of information that may affect treatment choice. Further, we must assume an underreporting of benign renal masses receiving expectant management because routine biopsy is not performed for active surveillance of renal masses. Because the NCDB is a cancer-focused registry, those small renal masses that are confirmed through biopsy or imaging findings as pathognomonic for benign tumors (eg, oncocytomas and angiomyolipomas) may not be included. The NCDB relies on a definitive clinical or pathologic diagnosis of renal cancer, which could lead to a potential underestimation of expectant management within our cohort. With this in mind, a cautious interpretation of the total proportion of expectant management is warranted. Although the article may underestimate the true prevalence of expectant management, we do feel this is a useful perspective on the prevalence of this practice using a national cancer registry. Furthermore, this particular source of bias should be nonselective; thus, the increase in proportion over the past decade is not affected by the above-mentioned limitations. As the NCDB does not capture ambulatory procedures, we have no data on practices that perform ablative therapies in an ambulatory setting. Patient preference and shared decision making are not captured in the NCDB, either, which is part of the American Urological Association guideline recommendation when considering active surveillance. Although a comorbidity score is available, other, more nuanced clinical factors, such as body mass index, GFR, and prior abdominal surgical history may affect the use of nonoperative management and are not available in the NCDB. Knowing how many patients initially on active surveillance received delayed surgical treatment would be interesting, but the necessary data are lacking in the NCDB. Focal ablation was introduced into guidelines after 2015, which makes it difficult to interpret trends before 2016 (34). Our results demonstrate an increase in the utilization of focal ablation following its inclusion in the guidelines.

There was an increase in the use of nonoperative management for small renal masses over the 10-year study period, with both expectant management and focal ablation being more frequently used in later study years. Our finding that expectant management was more commonly used, but focal ablation was less commonly used for Black patients is noteworthy. Plausible explanations include race-based differences in how physicians estimate competing risks and comorbidities as well as differences in the resources required for ablative therapies vs expectant management. The inclusion of Black race in calculations for GFR could also influence the differential uptake of these techniques either directly through differences in GFR calculation or indirectly if Black patients are more likely to harbor chronic renal disease through less aggressive management of renal risk factors. This finding warrants robust research efforts and policies to support equity in this critical area.

## Supplementary Material

pkad084_Supplementary_Data

## Data Availability

The data that support the findings of this study are openly available on the NCDB website (https://www.facs.org/quality-programs/cancer-programs/national-cancer-database/). The specific codes used for preprocessing and analysis of these data are available from the authors upon request, maintaining anonymity for peer review purposes. The authors commit to providing these resources promptly upon request for the purpose of evaluating the manuscript during the peer review process.
