# Supplementary material for: The national utilization of nonoperative management for small renal masses over 10 years
Source: JNCI Cancer Spectr. 2023 Oct 6;7(6):pkad084. doi: 10.1093/jncics/pkad084 (PMC10640883; doi:10.1093/jncics/pkad084)

Supplementary Figure 1: Proportion of Non-Operative Management within the National Cancer Database.

## Proportion of Expectant Management of Small Renal Masses

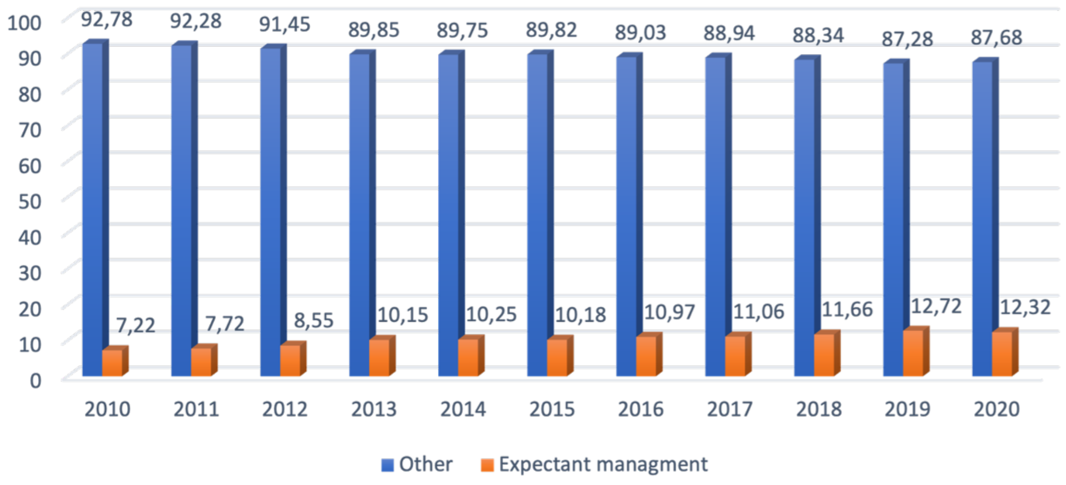


## Proportion of Ablation of Small Renal Masses

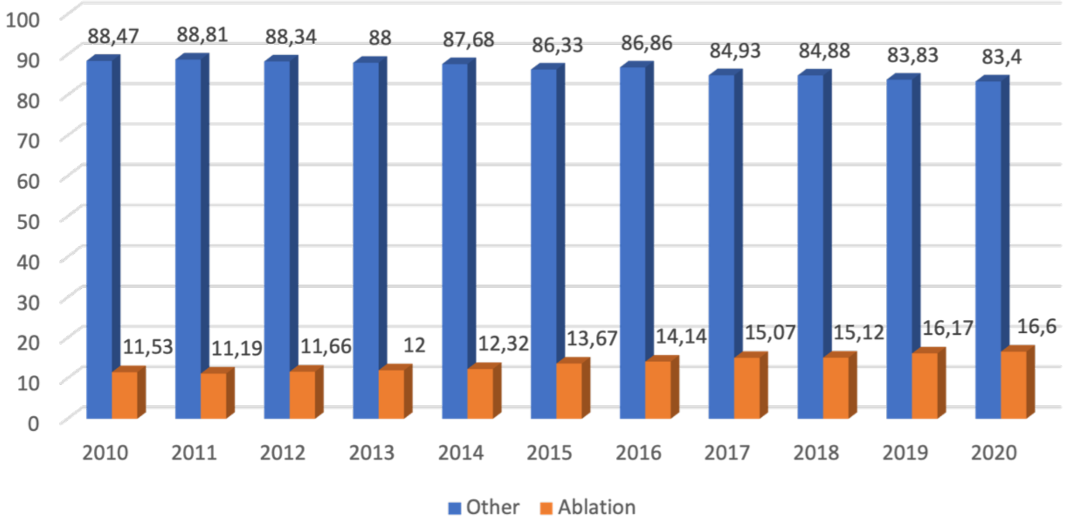


## Proportion of Non-Operative Management in Small Renal Masses

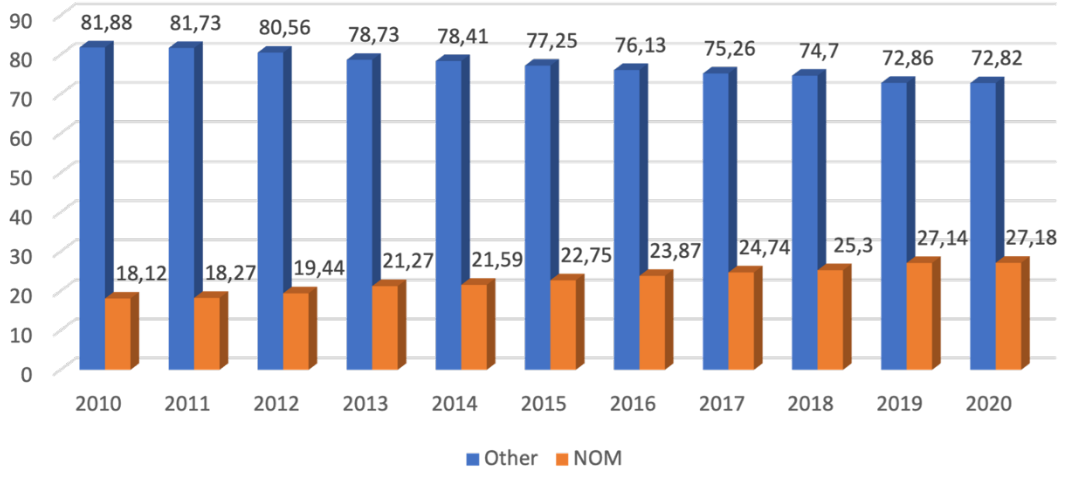

Supplement: pkad084_Supplementary_Data [file pkad084_supplementary_data.pdf]
